# Valproate-Enhanced Protocols for Alcohol Withdrawal Syndrome: A Brief Review and Retrospective Study of Efficacy and the Ability to Reduce Benzodiazepine Use

**DOI:** 10.3390/ph18060855

**Published:** 2025-06-08

**Authors:** Simone Pardossi, Alessandro Cuomo, Giacomo Gualtieri, Mario Pinzi, Giuditta Piumini, Andrea Fagiolini

**Affiliations:** 1 Department of Molecular Medicine, University of Siena School of Medicine, 53100 Siena, Italy; alessandro.cuomo@unisi.it (A.C.); mario.pinzi@student.unisi.it (M.P.); g.piumini@student.unisi.it (G.P.); 2 Department of Medicine, Surgery and Neuroscience, University of Siena, 53100 Siena, Italy; giacomo.gualtieri2@unisi.it

**Keywords:** alcohol withdrawal syndrome, valproate, substance use disorders, benzodiazepines, adjuvant therapy

## Abstract

**Background:** Although benzodiazepines are the first-line treatment for alcohol withdrawal syndrome (AWS), their use may pose significant risks, including oversedation and the potential for misuse, particularly in vulnerable populations such as individuals with alcohol use disorder. Valproate has been investigated as a potential adjunctive treatment for AWS. We first conducted a brief narrative review of the existing literature on valproate in AWS, identifying only a few relevant studies. We then performed a retrospective study to evaluate the effectiveness of valproate, administered orally (PO) or intravenously (IV), in combination with benzodiazepines for the treatment of AWS and associated anxiety symptoms. **Methods:** We retrospectively analyzed 72 inpatients treated for AWS with valproate (IV or PO) combined with benzodiazepines. Dosages of valproate, the type and daily dose of benzodiazepine, and any adverse effects were recorded. Withdrawal symptoms were assessed using the Clinical Institute Withdrawal Assessment for Alcohol Scale, Revised (CIWA-Ar) on days 1, 3, 5, and 7. Anxiety symptoms were evaluated using the Hamilton Anxiety Rating Scale (HAM-A) on days 1, 3, and 7. **Results**: The median daily benzodiazepine dose was 2.5 mg lorazepam-equivalents (IQR: 2.0–3.81 mg). Significant reductions in both CIWA-Ar and HAM-A scores were observed across all time points. Percentage reductions in both anxiety and withdrawal symptoms were significantly higher in the IV group. No serious adverse events occurred. **Conclusions**: Valproate appears to be an effective adjunctive treatment for AWS, providing symptom relief and enabling reduced benzodiazepine use. IV administration may offer more rapid clinical improvement. Larger prospective trials are warranted to confirm these findings.

## 1. Introduction

Alcohol withdrawal syndrome (AWS) is a severe, potentially life-threatening condition that can arise in individuals with chronic alcohol consumption after the abrupt cessation of or a substantial decrease in alcohol intake [1]. AWS presents a range of clinical symptoms, from mild signs of autonomic overactivity and tremors to life-threatening complications like seizures and delirium tremens (DT), and remains an important clinical challenge, seen in psychiatric and medical units [2,3]. Notably, DT occurs in approximately 3–5% of individuals undergoing alcohol withdrawal and, if left untreated, carries a mortality rate between 15% and 40% [4]. With appropriate medical intervention, this rate decreases significantly to approximately 1–4% [4]. Furthermore, alcohol use disorder (AUD) is a major contributor to global mortality. According to the World Health Organization, in 2019, alcohol consumption was responsible for approximately 2.6 million deaths worldwide, accounting for 4.7% of all global deaths [5]. The gold standard treatment for AWS is the use of benzodiazepines that serve as positive allosteric modulators of GABA-A receptors, reducing the hyperexcitable state caused by the abrupt cessation of alcohol use [2,6]. Although effective, benzodiazepines have many limitations, such as dose-dependent sedation, the risk of respiratory depression, and the risk of misuse, especially in patients with comorbid substance use disorders [7,8]. These concerns are leading to investigations of adjunctive pharmacologic strategies to reduce the benzodiazepine load, with improvement in clinical efficacy [9].

Among these alternatives, anticonvulsants have attracted attention due to their ability to both decrease excitatory neurotransmission and enhance GABAergic activity [10]. For instance, carbamazepine has demonstrated efficacy in reducing the severity of AWS; in fact, several trials have reported outcomes comparable to, or even better than, those achieved with benzodiazepines [11]. Nonetheless, its side effect profile and enzyme-inducing properties limit its use in certain populations [12]. Gabapentin and pregabalin have shown promise in initial studies, particularly in outpatient settings, but conclusive evidence is still lacking [13].

Valproate is an agent with broad-spectrum anticonvulsant action. Its mechanisms of action include the enhancement of GABAergic transmission, inhibition of voltage-gated sodium channels, and modulation of intracellular signaling cascades, including GSK-3β and histone deacetylases [14].

Clinical trials indicate that valproate is promising in the AWS population (Table 1). In a randomized trial by Lambie et al. [15], 49 detoxified inpatients were enrolled, and the study demonstrated that valproate significantly reduced seizure activity and hastened the resolution of physical withdrawal symptoms, despite fewer patients receiving adjunctive sedative medications. In an open-label study by Rosenthal et al. [16], valproate and phenobarbital were found to be equally effective in reducing both subjective and objective signs of withdrawal. However, phenobarbital was associated with lower reported levels of hostility. Importantly, neither group experienced seizures nor serious adverse events related to withdrawal. Reoux et al. [17] conducted a double-blind, placebo-controlled trial involving 36 inpatients, showing that valproate significantly attenuated the progression of withdrawal symptoms and reduced the need for rescue benzodiazepines (oxazepam), particularly in patients who remained symptomatic beyond the first 12 h. Longo et al.’s study further highlighted valproate’s potential utility in relapse prevention. They reported that patients receiving valproate exhibited better symptom control, and those maintained on valproate for six weeks achieved higher abstinence rates at follow-up. An open-label pilot study by Myrick et al. [18] compared divalproex sodium (500 mg TID) to symptom-triggered lorazepam in 11 inpatients with uncomplicated alcohol withdrawal. Both groups received lorazepam as needed based on CIWA-Ar scores >6, but patients in the divalproex group required significantly lower total doses. The divalproex group also showed a faster and more stable reduction in withdrawal symptoms. Divalproex was well tolerated, with no major adverse effects or liver function abnormalities.

Other studies have investigated the use of valproate in the treatment of alcohol withdrawal symptoms. For example, a pilot study [19] evaluated an aggressive oral loading dose of divalproex sodium in three patients with moderate alcohol withdrawal, showing that therapeutic valproate plasma levels could be safely achieved within 2–6 h and were associated with a clinical improvement in withdrawal symptoms, in some cases without the need for benzodiazepines. A case series [20] reported two cases of patients with mania and comorbid alcohol withdrawal treated with oral loading doses of divalproex (20 mg/kg/day). One patient required only two 2 mg doses of lorazepam; the other, after divalproex initiation, required no further benzodiazepines. In both cases, manic symptoms improved within 72 h, and CIWA-Ar scores for alcohol withdrawal dropped rapidly, reaching zero in one case.

Nevertheless, the number of well-controlled, large-scale trials remains limited, and further research is needed to clarify valproate’s role within standard clinical protocols. Moreover, to date, no comparative studies in AWS have examined different routes of valproate administration—namely, intravenous (IV) versus oral (PO)—and their differential impact on symptom trajectory and benzodiazepine use remains poorly characterized. Moreover, the specific contribution of valproate to the modulation of anxiety symptoms during AWS is a topic of growing interest, given the bidirectional relationship between AUD and anxiety disorders, and the prominent role of anxiety in the symptomatology of withdrawal [21]. In this context, the present study aimed to retrospectively assess the clinical effectiveness of valproate, administered via either intravenous or oral routes, in combination with benzodiazepines, for the treatment of AWS.

The primary aim of our study was to assess whether the combination of valproate and benzodiazepines could be effective in treating alcohol withdrawal symptoms and anxiety symptoms in hospitalized patients. The secondary aim was to compare the efficacy in withdrawal symptoms of PO versus IV valproate, both in combination with benzodiazepines, to understand whether there are differences in the efficacy of these treatments in managing withdrawal and anxiety symptoms.

## 2. Results

### 2.1. Descriptive Statistics

The sample consisted of 72 patients (62.5% males), with a mean age of 45.65 years (SD = 14.04) and a mean BMI of 24.21 (IQR: 21.45–26.92). The most common route of administration for valproate was IV in 51.4% of cases, followed by PO in 48.6%. In the OS group, females represented 34.3% of the sample, with a mean age of 45.7 years (SD = 14.6), while in the EV group, females accounted for 40.5%, with a mean age of 45.6 years (SD = 13.6). The PO formulation was administered as extended-release tablets containing sodium valproate and valproic acid.

### 2.2. Valproate and Benzodiazepine Dosages

The median daily benzodiazepine lorazepam-equivalent dosage was 2.5 mg (IQR: 2.0–3.81). The distribution was not normal (Shapiro–Wilk *p* < 0.001) and showed right-sided skewness. Lorazepam was the most frequently used benzodiazepine (62.0%), followed by delorazepam (32.4%), clonazepam (2.8%), alprazolam (1.4%), and diazepam (1.4%).

Valproate administration dosages were as follows: On day 1, the intravenous (IV) group had a median dosage of 800 mg (25% = 800, 75% = 800), and the oral solution (OS) group had a median of 800 mg (25% = 500, 75% = 1000). On day 3, IV dosage was 800 mg (25% = 800, 75% = 800), and OS dosage was 1000 mg (25% = 600, 75% = 1000). On day 5, IV dosage was 800 mg (25% = 800, 75% = 1000), and OS dosage was 1000 mg (25% = 800, 75% = 1000). Mean blood valproate concentrations increased from 49.40 mg (SD = 14.77) on day 3 to 59.56 mg (SD = 14.83) on day 5.

### 2.3. HAM-A Scale

**Figure 1:**
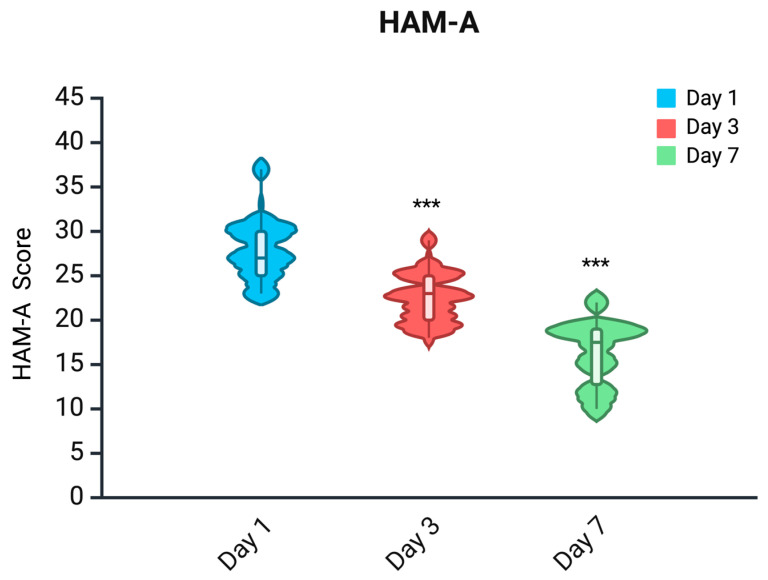
Distributions of Hamilton Anxiety Rating Scale (HAM-A) scores. ***: *p* < 0.001 (statistically significant reduction).

Shapiro–Wilk tests revealed a non-normal distribution for HAM-A scores at all time points (*p* < 0.05). The Friedman test showed a significant overall difference across day 1 (T0), day 3 (T1), and day 7 (T2) (χ^2^ = 141.09, *p* < 0.001). Post hoc Wilcoxon signed-rank tests confirmed significant pairwise reductions in HAMA scores (Bonferroni-corrected *p* < 0.001 for all comparisons). Median values decreased from 27 (IQR: 25–30) at T0 to 23 (IQR: 20–25) at T1, and to 17.5 (IQR: 12.75–19) at T2 (Figure 1).

### 2.4. CIWA-Ar Scale

**Figure 2:**
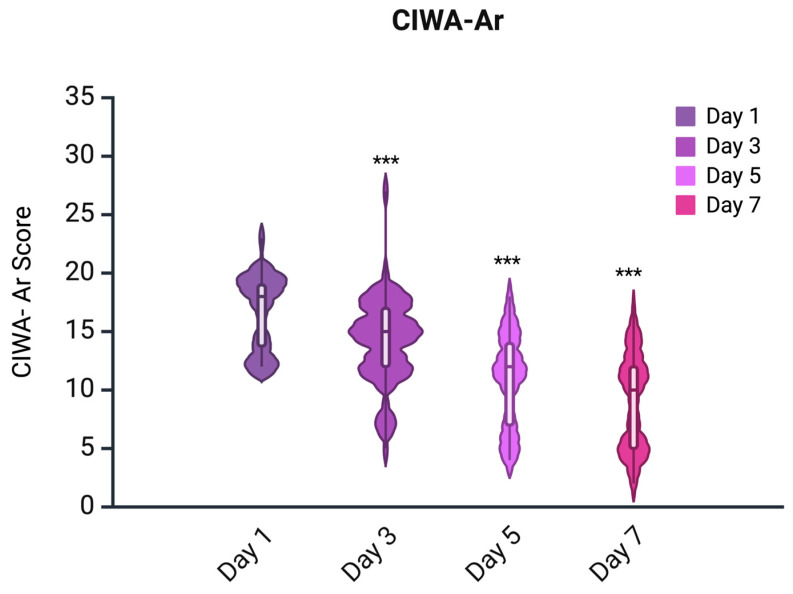
Distributions of Clinical Institute Withdrawal Assessment for Alcohol Scale (CIWA-Ar) scores. ***: *p* < 0.001 (statistically significant reduction).

CIWA-Ar scores were non-normally distributed at all time points, as confirmed by the Shapiro–Wilk test (*p* < 0.05). The Friedman test showed a significant overall reduction in CIWA-Ar scores across the four assessment days (day 1, 3, 5, and 7) (χ^2^ = 197.78, *p* < 0.001). Pairwise comparisons using the Wilcoxon signed-rank test indicated statistically significant improvements between all time points (*p* < 0.001, Bonferroni-corrected) (Figure 2).

### 2.5. Comparison of PO-EV

A between-group comparison showed that at baseline (T0), patients in the EV group had significantly higher HAM-A scores than those in the PO group (median 30 vs. 27; *p* < 0.001). No statistically significant difference was observed at day 3 (T1) between the two groups (*p* = 0.932), whereas at day 7 (T2), HAMA scores were significantly lower in the EV group compared to PO (median 15 vs. 19; *p* = 0.015).

Absolute changes in HAMA scores were calculated to assess treatment efficacy over time. EV treatment resulted in significantly greater reductions than PO at both intervals (median Δ = −6 vs. −4 at day 3; *p* < 0.001; and −7 vs. −4 at day 7; *p* = 0.003). Relative percentage reductions were also significantly greater in the EV group compared to PO. At day 3, the median percentage decrease was −20.0% in the EV group vs. −14.81% in the PO group (*p* < 0.01); at day 7, the reduction reached −28.57% vs. −17.39%, respectively (*p* < 0.01).

Regarding CIWA-Ar, no significant differences were found between the PO and EV groups at baseline (day 1, *p* = 0.36) or day 3 (*p* = 0.45). At day 5, CIWA-Ar scores were lower in the EV group compared to PO, but the difference (*p* = 0.03) did not remain significant after Bonferroni correction. At day 7, CIWA-Ar scores were significantly lower in the EV group (*p* < 0.01).

The absolute change in CIWA-AR-AR scores over time was compared between groups. The EV group showed a greater reduction in scores from day 1 to day 3 (median −4.0 vs. −2.0; *p* < 0.01), from day 3 to day 5 (–3.0 vs. −2.0; *p* = 0.02), and from day 5 to day 7 (–2.0 vs. 0.0; *p* < 0.01). However, the difference from day 3 to day 5 did not remain significant after correction for multiple comparisons.

Similarly, the relative percentage changes showed greater improvement in the EV group. At day 3, the median percentage reduction was −20.0% in the EV group versus −10.53% in the PO group (*p* < 0.01). At day 5 and day 7, the EV group continued to show significantly larger percentage reductions compared to PO (*p* < 0.01 for all comparisons).

## 3. Discussion

Of the total sample, 62.5% of patients were male. This finding is consistent with known gender differences in the epidemiology of alcohol use disorder, which is more frequently diagnosed in men than in women [22,23]. The mean age of our sample was approximately 45 years. This figure should be contextualized within the setting of our study, which was conducted in an adult psychiatry service and therefore naturally includes an older clinical population. Notably, heavy alcohol use has been shown to be particularly frequent among middle-aged and older adults (55–70 years), although younger individuals may present more frequently with alcohol use disorder [24].

In our sample of hospitalized patients, valproate was administered either intravenously or orally. In both cases, we observed a statistically significant reduction in anxiety symptoms as early as day 1 through day 3. Naturally, being admitted to a psychiatric ward may itself provide some reassurance to the patient. However, it is important to underline that patients had completely stopped consuming alcohol during this period. Despite this, not only did they not experience a worsening of anxiety symptoms, but they actually showed a clear improvement with the treatment.

The amelioration of anxiety symptoms might be related to the use of both benzodiazepines and valproate. The role of benzodiazepines in anxiety symptoms has indeed been largely studied [25]. In addition to its well-recognized role as an anticonvulsant and mood stabilizer, valproate has also been considered a potential agent to treat anxiety symptoms [26,27].

Moreover, it is fundamental to point out the relationship between anxiety symptoms and alcohol withdrawal: in fact, the former is one of the main manifestations of the latter. Anxiety is also one of the main criteria for CIWA-Ar scoring [28]. Regarding this, we observed a statistically significant reduction at all time points, indicating a progressive reduction in anxiety (as measured by HAM-A), accompanied by a progressive reduction in withdrawal symptoms as well.

Thus, both anxiety symptoms and withdrawal symptoms improved within the very first two days, and this trend progressively continued over the following days, up to day 7.

Benzodiazepines are considered the primary pharmacological treatment for AWS [6]. However, patients with substance use disorders, such as AUD, might be inclined to overuse benzodiazepines [7], especially considering that 30–40% of patients with AUD also develop benzodiazepine misuse [29]. Therefore, although benzodiazepines are a cornerstone in the treatment of AWS, their use should be limited after the acute phase [7]. This is more feasible if, during the acute phase, benzodiazepines are used at the minimum effective dose [30].

Most trials on AWS report lorazepam dosages ranging from 6 to 12 mg [31,32,33]. In our cohort, a median dose of 2.5 mg, combined with valproate, was sufficient to achieve a meaningful reduction in withdrawal and anxiety symptoms. This result is consistent with previously cited evidence by Myrick et al. [18], who showed that the use of divalproex sodium not only enhanced clinical efficacy but also reduced the need for benzodiazepines. Also, in the case series by Hammer et al. [20], two patients with alcohol withdrawal symptoms were treated with a combination of valproate and lorazepam, with one patient requiring only 2 mg of lorazepam and the other completing treatment without the need for any benzodiazepines. Regarding valproate, even though there is no clearly established recommended blood level for psychiatric disorders, the range of 50–100 mcg/mL is typically used for epilepsy, and some studies suggest a therapeutic range of 50–125 mcg/mL in mania [34]. Our cohort’s mean valproate blood level on day 1 was 59.56 mcg/mL (SD = 14.83).

We also compared patients treated with IV valproate and those treated with PO valproate. To our knowledge, no other studies have specifically investigated the role of IV valproate in AWS. However, IV valproate has been studied in the context of agitation and mania, and its calming, anxiolytic, and anti-manic effects have been assessed in a few studies, although the evidence remains sparse [35,36].

In our cohort, we observed higher HAM-A scores in the group treated with IV valproate, likely reflecting the fact that clinicians selected the IV formulation for patients with more severe symptomatology. Interestingly, the reduction in anxiety symptom severity was greater in the IV group compared to the PO group. A similar trend was observed for withdrawal symptoms, as measured by CIWA-Ar, with a greater reduction in the IV group.

These observations may suggest a potentially faster and more effective action of the IV formulation. As this is the first study investigating the use of intravenous valproate in patients with AWS, it is difficult to determine why the IV formulation appeared more effective than the oral one. One hypothesis is that intravenous administration delivers the full dose of the drug immediately and with complete bioavailability, thus being potentially more efficacious in patients with impaired absorption. Additionally, the supportive effect of intravenous hydration and a possible placebo effect associated with IV administration may also have contributed to the observed difference.

Retrospectively, we were able to exclude the occurrence of any serious adverse drug reactions during the inpatient treatment period. However, we cannot rule out the possibility that some mild side effects may have occurred and resolved spontaneously without being systematically recorded in the medical charts. Such effects might include gastrointestinal discomfort, mild tremors, transient sedation, or headache—commonly reported adverse effects associated with valproate use.

## 4. Materials and Methods

A retrospective chart review was conducted on 72 patients admitted to the University of Siena Medical Center between January 2019 and January 2024 for alcohol withdrawal syndrome (AWS). Inclusion criteria were hospitalization for AWS and treatment with valproate in combination with benzodiazepines. Exclusion criteria included a history of hypersensitivity to valproic acid, significant medical or neurological comorbidities, or pregnancy and breastfeeding. Patients were treated with valproate either in IV or PO formulation, according to clinical judgment, and benzodiazepine administration was tailored based on symptom severity. The type and dosage of benzodiazepine used (e.g., lorazepam, delorazepam, clonazepam) were documented.

We collected sociodemographic data (age, sex, BMI) and clinical variables including medication regimens. Symptom severity was assessed using validated clinical rating scales. Specifically, CIWA-Ar was administered on days 1, 3, 5, and 7 of hospitalization to evaluate the progression of alcohol withdrawal symptoms. Additionally, anxiety symptoms were measured using the Hamilton Anxiety Rating Scale (HAM-A) on days 1, 3, and 7. Relevant adverse effects were also recorded, if present. All assessments were performed by trained clinicians as part of routine clinical monitoring.

The normality of the variables was tested using the Shapiro–Wilk test. As most variables did not follow a normal distribution, non-parametric tests were applied. Between-group comparisons were conducted using the Mann–Whitney U test, while within-group comparisons over time were assessed using the Wilcoxon signed-rank test. Categorical variables were analyzed using the chi-square test. All tests were two-tailed, and a *p*-value < 0.05 was considered statistically significant.

To compare treatment response between the two groups (EV vs. PO), the Mann–Whitney U test was used at each time point. In addition to raw scores, we calculated both absolute and percentage change scores between the time points to assess the magnitude and trajectory of clinical improvement.

Statistical analyses were carried out using R software (version 4.3.1).

This study received ethical approval from the institutional review board (protocol number 20931). Prior to data collection, all participants provided written informed consent after receiving a comprehensive explanation of the study’s objectives and procedures. The research was conducted in full compliance with the principles outlined in the Declaration of Helsinki and adhered to Good Clinical Practice (GCP) guidelines.

## 5. Conclusions

In conclusion, the use of valproate—administered both orally and intravenously—played a key role in managing withdrawal and anxiety symptoms in our cohort of hospitalized patients with AWS, and importantly, it allowed, in our cohort, for the use of benzodiazepines at relatively low doses. This strategy may be particularly relevant in minimizing the risks associated with benzodiazepines, especially in the long term and in populations with a high vulnerability to dependence, such as those with AUD.

### Limitations

There are some limitations of this study that need to be addressed. First, the retrospective design limits the ability to establish causal relationships between treatment and clinical outcomes. The sample represented a group of patients treated in clinical practice, which may limit the generalizability of findings. Because treatment assignment (PO vs. IV valproate) was determined by clinical judgment rather than randomization, the between-group differences observed may reflect baseline differences in symptom severity rather than true treatment effects. Second, the sample size was modest, particularly when divided into subgroups. Third, there was no control condition to determine the extent to which valproate alone contributed to symptom improvement; given the established efficacy of benzodiazepines in AWS, the specific role of valproate could not be isolated. Although our results suggest that valproate may have allowed for lower benzodiazepine dosing than would otherwise be required, only a prospective controlled trial could confirm this.

Larger, prospective, and potentially multicenter studies are warranted to better define the role of valproate—especially in its intravenous form—in the acute management of AWS and related anxiety symptoms.

## Figures and Tables

**Table 1:** Trials on the use of valproate for alcohol withdrawal syndrome. CIWA-Ar: Clinical Institute Withdrawal Assessment for Alcohol Scale, Revised; MSSA: Minnesota Substance Abuse Survey; ASQ: Alcohol Symptom Questionnaire.

Study	Groups	Scale	Result
Lambie et al., 1980 [15]	Placebo (n = 27) vs. Valproate (n = 22)	3 grades of symptoms: Grade 0: no symptoms; Grade 1: physical symptoms but clear consciousness; Grade 2: physical symptoms with clouding of consciousness	Faster amelioration of withdrawal symptoms with valproate
Longo et al., 2002 [15]	Benzodiazepine n = 7) vs. Divalproex Sodium (n = 5) 5 Days vs. Divalproex Sodium 5 Days + 6-Week Maintenance (n = 5)	CIWA-Ar	Greater reduction in CIWA-Ar in valproate groups; Abstinence better maintained in the 6-week valproate maintenance group
Reoux et al., 2001 [17]	Placebo (n = 18) vs. Valproate (n = 18)	CIWA-Ar	Greater reduction in withdrawal symptoms observed in the valproate group
Rosenthal et al., 1998 [16]	Phenobarbital (n = NR) vs. Valproate (n = NR)	MSSA; ASQ	No significant difference between the two groups in the improvement in withdrawal symptoms
Myrick et al., 2000 [18]	Divalproex Sodium + Lorazepam (n = 6) vs. Lorazepam (n = 5)	CIWA-Ar	

## Data Availability

The original contributions presented in this study are included in the article. Further inquiries can be directed to the corresponding authors.

## Abbreviations

The following abbreviations are used in this manuscript:

AWS	Alcohol Withdrawal Syndrome
IV	Intravenous
OS	Oral
CIWA-Ar	Clinical Institute Withdrawal Assessment for Alcohol Scale, Revised
HAM-A	Hamilton Anxiety Rating Scale
DT	Delirium Tremens
GABA-A	Gamma-Aminobutyric Acid Type A Receptor
GSK-3β	Glycogen Synthase Kinase 3 Beta
MSSA	Minnesota Substance Abuse Survey
ASQ	Alcohol Symptom Questionnaire
GCP	Good Clinical Practice
